# Fine droplet misting and stage-specific nutrient formulation enhance bed-area biomass productivity and nutrient solution use efficiency in closed-loop aeroponic *Centella asiatica*

**DOI:** 10.3389/fpls.2026.1892738

**Published:** 2026-07-23

**Authors:** Ju Young Lee, Jeong Do Kim, Jai-Eok Park

**Affiliations:** Korea Institute of Science and Technology (KIST), Gangneung Institute of Natural Products, Smart Farm Convergence Research Center, Gangneung, Republic of Korea

**Keywords:** aeroponics, biomass yield, *Centella asiatica*, droplet size, dynamic nutrient formulation, misting interval, nozzle, solution-use efficiency

## Abstract

Closed-loop aeroponics exposes suspended roots to repeated pulses of nutrient solution and humid air, so nozzle and misting settings shape the root-zone water, nutrient, and gas environment. We evaluated how root-zone droplet delivery and stage-specific ion management affected growth and nutrient solution-use efficiency of *Centella asiatica* in a natural-light greenhouse. Six nozzle classes were characterized by laser diffraction, and three misting schedules were screened using shoot dry-mass production efficiency. A separate bed-level experiment compared fresh and dry biomass under four operationally feasible nozzles, while a nutrient-management experiment compared fixed Hoagland, EC-adjusted Hoagland, and a stage-specific formulation using shoot and root biomass, leaf and runner number, SPAD, canopy cover, and solution-use efficiency. The plantable bed area was 63.9 m² within a 165 m² greenhouse, at a density of approximately 16–25 plants m^−^². VF and F treatments achieved the highest dry-mass production efficiency, and the 5 min × 288 events day^−^¹ schedule performed best across nozzle classes. At 53 days after transplanting (DAT), VF produced the greatest fresh and dry biomass per bed area within the tested C–M–F–VF range. The stage-specific formulation produced greater shoot and root biomass, canopy cover, and solution-use efficiency than either Hoagland treatment. Reservoir corrections were greatest for nitrate during leaf expansion, potassium during biomass accumulation, and Ca, Fe, and B during late maturation. Taken together, the complementary experiments associated root-zone delivery and stage-specific nutrient supply with coordinated whole-plant growth and resource-use responses. Because root-surface wetness, oxygen availability, root respiration, and tissue ion accumulation were not measured directly, nozzle effects should be interpreted as integrated root-zone treatment effects rather than effects of droplet diameter alone. Multi-season factorial experiments with direct physiological measurements are needed to confirm the underlying mechanisms.

## 1 Introduction

*Centella asiatica* (L.) Urban is a stoloniferous medicinal and cosmetic crop whose creeping habit, runner-based propagation, and relatively short canopy make it suitable for controlled-environment and vertical-farm production. Hydroponic studies have shown that *C. asiatica* can be cultivated in soilless systems and that EC, light, growth stage, and nutrient formulation influence biomass formation and centelloside accumulation (Prasad et al., 2012; Shawon et al., 2023, 2025; Harakotr et al., 2024; Lee et al., 2025). For production-scale aeroponics, stable and repeatable biomass production per unit cultivation area and per unit volume of nutrient solution is an important prerequisite.

Aeroponics differs from NFT and DFT because roots are suspended in a humid air space and receive nutrient solution as droplets. In water-based cultivation, roots must acquire water, dissolved ions, and oxygen simultaneously; inadequate irrigation rate or spatial distribution can restrict one resource even when bulk-solution composition appears adequate (Blok et al., 2017). Aeroponics increases root exposure to air but provides little hydraulic buffering, so delivery frequency, droplet spectrum, root-zone temperature and humidity, and solution recirculation can affect root hydration, gas exchange, nutrient contact, and whole-plant growth (Lakhiar et al., 2018; Eldridge et al., 2020; Li et al., 2018; Tunio et al., 2021, 2022). Nozzle properties are therefore biologically relevant because they help define the physical environment at the root surface.

Closed-loop operation adds another layer of complexity. EC indicates total ionic strength but does not reveal shifts in individual ion ratios. Ion-specific monitoring and adaptive replenishment have therefore been proposed to improve EC-based management in closed hydroponic systems (Sonneveld and Voogt, 2009; Savvas and Gruda, 2018; Gang et al., 2025). Preliminary monitoring in the present system indicated comparatively large K correction requirements during vegetative growth, large nitrate corrections during canopy expansion, relatively stable Ca corrections, and increasing Fe and B corrections during new-leaf development. These observations motivated a stage-specific formulation aimed at biomass production rather than maximum secondary-metabolite concentration.

The central plant-science question was how root-zone solution delivery and stage-dependent ion availability were associated with growth and resource use in *C. asiatica*. Nozzle measurements characterized the imposed delivery treatments rather than serving as standalone engineering endpoints. We evaluated (i) whether droplet-delivery treatments were associated with shoot dry-mass production efficiency and bed-area biomass, (ii) whether operation under the 5 min × 288 events day^−^¹ schedule was accompanied by a stable, high-humidity root zone and efficient biomass production, (iii) whether stage-specific nutrient formulation improved shoot and root biomass, leaf and runner formation, SPAD, canopy development, and solution-use efficiency beyond fixed or EC-adjusted Hoagland management, and (iv) how maintenance, seasonal light variation, and bed-level replication constrained interpretation. Because nozzle class simultaneously changes droplet size, spray angle, flow distribution, and clogging risk, nozzle effects were interpreted as integrated root-zone treatment effects unless individual components were tested independently.

## 2 Materials and methods

### 2.1 Plant material and closed-loop aeroponic system

**Figure 1:**
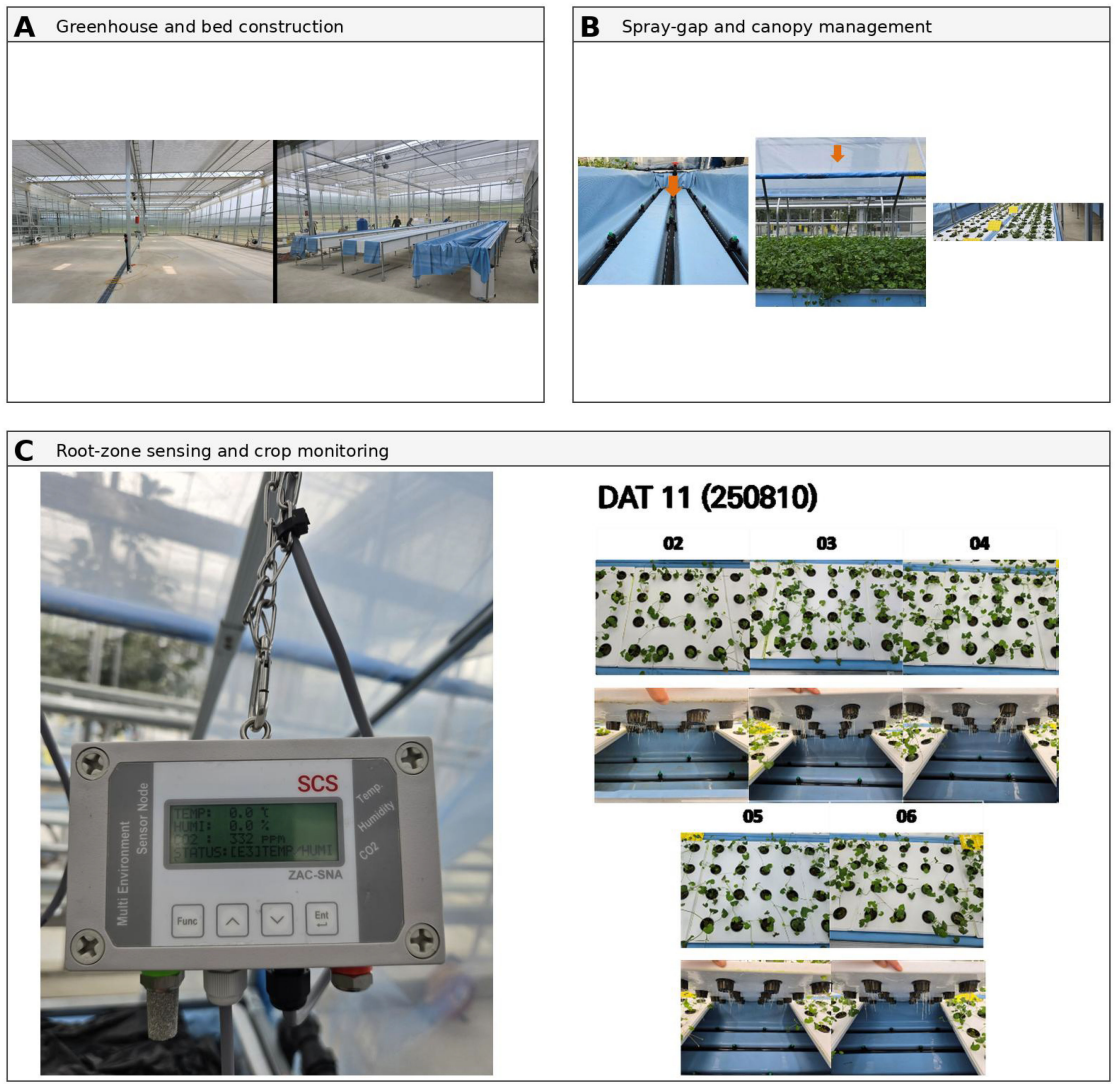
Field views of the closed-loop aeroponic *C. asiatica* system at the Hoenggye greenhouse. Panel **(A)** shows greenhouse and bed construction; panel **(B)** shows system modifications, including central-nozzle addition, winter vinyl-tunnel installation, and crop-management setup; panel **(C)** shows root-zone environmental sensing and crop monitoring. These images document the hardware and monitoring context of the production-oriented experiments.

Uniform *C. asiatica* plantlets were established in a closed-loop aeroponic system at the KIST AI Hoenggye demonstration greenhouse in Daegwallyeong-myeon, Pyeongchang-gun, Gangwon-do, Republic of Korea (hereafter, the Hoenggye greenhouse). The natural-light, multi-span facility was covered with fluorine film and used for aeroponic cultivation trials. The total greenhouse floor area was 165 m², whereas the plantable bed surface area was 63.9 m² (three beds × 1.42 m × 15.0 m). Planting density ranged from approximately 16 to 25 plants m^−^² of bed surface, reflecting practical bed filling and runner-based canopy establishment. All area-normalized yields and crop-cycle extrapolations therefore use plantable bed surface area unless otherwise stated. The greenhouse scale, bed-area basis, and experimental-unit definition are summarized in **Table 1**. The light environment comprised transmitted natural sunlight rather than a fixed artificial-light set point. Seasonal internal PPFD and DLI ranges are summarized in **Table 1B**. Date-specific canopy-level PPFD or DLI records were not available for every crop cycle; this limitation should be considered when comparing cycles (Both et al., 2015; Torres and Lopez, 2011; Faust and Logan, 2018). Plants were held in planting panels above a covered root chamber. Nutrient solution was pumped from a recirculating reservoir to suspended roots through single-fluid misting nozzles and returned to the reservoir after root-zone contact. Sensors for root-zone temperature and relative humidity were mounted below the planting panel, and reservoir EC and pH were logged throughout the crop cycle. Representative views of the greenhouse, aeroponic system, root-zone sensing, and crop monitoring are shown in **Figure 1**.

**Table 1:** Demonstration greenhouse scale, bed-area basis, and experimental-unit definition.

Item	Value used in this manuscript	Interpretation for yield analysis
Total greenhouse floor area	165 m²	Includes crop beds, aisles, equipment, and working space
Plantable bed surface area	63.9 m²	3 beds × 1.42 m × 15.0 m; all g m^−^² values are based on bed surface area
Area-normalization basis	g m^−^² bed area	Not normalized to the total 165 m² greenhouse floor area unless explicitly stated
Independent experimental unit	Bed-level replicate	Plant or subsample measurements were averaged at the bed level before analysis
Light environment	Natural sunlight in the Hoenggye greenhouse (see **Table 1B**)	Use crop-cycle PPFD/DLI as metadata or covariate
Planting density	Approximately 16–25 plants m^−^² of plantable bed surface	Used to interpret canopy closure, per-bed plant number, and production scaling

**Table 1B:** Observed seasonal internal light environment of the Hoenggye greenhouse used to contextualize natural-light aeroponic *C. asiatica* production.

Crop/light period	Cultivation period	External environment level	Observed internal PPFD	Daily mean DLI
Establishment	Late April–early May	Medium-high	180–320 µmol m^−^² s^−^¹	7–12 mol m^−^² day^−^¹
Early vegetative growth	May	High	250–450 µmol m^−^² s^−^¹	10–16 mol m^−^² day^−^¹
Vigorous growth	June	Very high; long photoperiod	350–600 µmol m^−^² s^−^¹	14–22 mol m^−^² day^−^¹
Rainy/high-temperature period	July	Highly variable	180–450 µmol m^−^² s^−^¹	7–16 mol m^−^² day^−^¹
Late vegetative growth	August	High to medium	220–500 µmol m^−^² s^−^¹	9–18 mol m^−^² day^−^¹
Autumn crop period	September	Medium	180–380 µmol m^−^² s^−^¹	7–13 mol m^−^² day^−^¹
Low-light transition	October	Declining	120–280 µmol m^−^² s^−^¹	5–10 mol m^−^² day^−^¹
Winter low-light period	November–February	Low	60–200 µmol m^−^² s^−^¹	2–7 mol m^−^² day^−^¹

### 2.2 Nozzle characterization and misting scenarios

Six nozzle classes (XC, UC, C, M, F, and VF) were characterized to describe the imposed root-zone delivery treatments. SMD, VMD, x10, x50, x90, spray angle, and flow rate were measured with a laser-diffraction particle-size analyzer at 4.0 bar and a measurement distance of 500 mm. These measurements described the liquid-delivery environment and were not treated as biological endpoints. For production-efficiency screening, each nozzle was tested under three misting schedules: 3 min × 480 events day^−^¹, 5 min × 288 events day^−^¹, and 6 min × 240 events day^−^¹. Production efficiency was calculated as shoot dry mass divided by the daily nutrient-solution spray-volume index and expressed as g DW L^−^¹. The screening dataset comprised three replicate observations per nozzle × schedule combination.

### 2.3 Bed-area biomass experiment

Based on the screening results and operational feasibility, C, M, F, and VF were selected for bed-area biomass testing. UC and XC were not included because their larger droplet spectra and operational requirements were less suitable for the target commercial configuration. Fresh biomass, dry biomass, and dry-matter percentage were measured at 34 and 53 DAT, and daily fresh-biomass gain between harvests was calculated. Four bed-level replicates were evaluated per nozzle at each harvest. When multiple plants were measured within a bed, they were treated as subsamples and averaged before analysis to avoid pseudoreplication; inference was based on bed-level units (Hurlbert, 1984). Fresh and dry biomass were expressed per square meter of plantable bed surface.

### 2.4 Stage-specific nutrient formulation and validation

Three nutrient-management treatments were compared in a plant-response validation experiment: a fixed Hoagland formulation, an EC-adjusted Hoagland formulation, and a stage-specific formulation. The fixed treatment used the same recipe throughout the crop cycle (Hoagland and Arnon, 1950). The EC-adjusted treatment restored total ionic strength while retaining the Hoagland ion ratios. The stage-specific treatment used crop-stage targets for nitrate-N, ammonium-N, P, K, Ca, Mg, S, Fe, Mn, Zn, Cu, B, and Mo. NO_3_–N and NH_4_–N are reported as mg N L^−^¹; all other nutrients are reported as elemental mg L^−^¹. Plant-response variables were shoot fresh and dry weight, leaf and runner number, root dry weight, SPAD, canopy cover, and solution-use efficiency. The validation dataset comprised four bed-level replicates per nutrient-management treatment. The stage-specific nutrient targets and corresponding 3-day correction values are summarized in **Table 2**.

**Table 2:** Stage-specific nutrient targets and 3-day correction values used for dynamic ion management.

Stage	Timing	EC target (dS m^−^¹)	pH target	NO_3_–N	K	Ca	Mg	Fe	B	3-day correction (mg L^−^¹)
Establishment	1–2 weeks	1.2–1.4	5.8–6.2	145	190	160	38	2.00	0.33	K 22.8; NO_3_–N 13.1; Ca 8.0
Leaf expansion	3–6 weeks	1.6–1.8	5.8–6.2	183	265	180	45	2.50	0.45	K 47.7; NO_3_–N 25.6; Ca 12.6
Biomass accumulation	6–10 weeks	1.7–1.9	5.8–6.2	168	305	190	52	2.50	0.50	K 61.0; NO_3_–N 20.2; Ca 13.3
Late maturation	>10 weeks/extended culture	1.5–1.7	5.8–6.3	148	255	210	48	3.00	0.60	K 35.7; NO_3_–N 13.3; Ca 25.2

Complete macro- and micronutrient targets, pre-replenishment concentrations, and calculated corrections are provided in **Supplementary Table 4**. The late-maturation formulation was intended for culture beyond 10 weeks and requires separate validation when the harvest window is extended.

### 2.5 Root-zone environment and nutrient correction monitoring

During operation under the 5 min × 288 events day^−^¹ schedule, root-zone temperature, root-zone relative humidity, line pressure, reservoir EC, and pH were recorded at 7, 21, 42, and 53 DAT. Nutrient-solution chemistry was evaluated using the stage-specific target after replenishment, the concentration before replenishment, and the calculated correction over a 3-day interval. The calculated correction was the concentration required to restore the reservoir to its stage-specific target after the observed decline. For **Figure 7**, the relative correction requirement was calculated as 100 × [(target after replenishment − concentration before replenishment)/target after replenishment]. This percentage represents the fraction of the stage-specific reservoir target restored after 3 days; it is not a direct measure of nutrient uptake or tissue accumulation. Root-zone RH was treated as a chamber-level environmental descriptor rather than a direct measure of root-surface liquid films or oxygen flux.

### 2.6 Statistical analysis

The bed, rather than the individual plant, was the independent experimental unit. Plant measurements within a bed were treated as subsamples and averaged to obtain one bed-level value, thereby avoiding pseudoreplication. The bed-area biomass trial used four bed-level replicates per nozzle, and the nutrient-management validation used four bed-level replicates per treatment. For production-efficiency screening, nozzle class, misting schedule, and their interaction were evaluated by two-way ANOVA when model assumptions were met; otherwise, Welch-type or nonparametric sensitivity analyses were used (Montgomery, 2017; Piepho and Edmondson, 2018). Bed-area biomass and nutrient-management endpoints were analyzed by one-way ANOVA followed by Tukey HSD for each harvest or response variable. Linear regression and Pearson correlation were used only for exploratory analyses. Correlations based on nozzle-level means or stage-level monitoring points were interpreted descriptively because these aggregated observations did not constitute additional independent experimental units. Natural-light PPFD and DLI were treated as environmental metadata and should be used as covariates or blocking factors in future multi-season trials. Statistical significance was evaluated at α = 0.05.

## 3 Results

### 3.1 Root-zone droplet-delivery treatments altered dry-mass production efficiency

**Figure 2:**
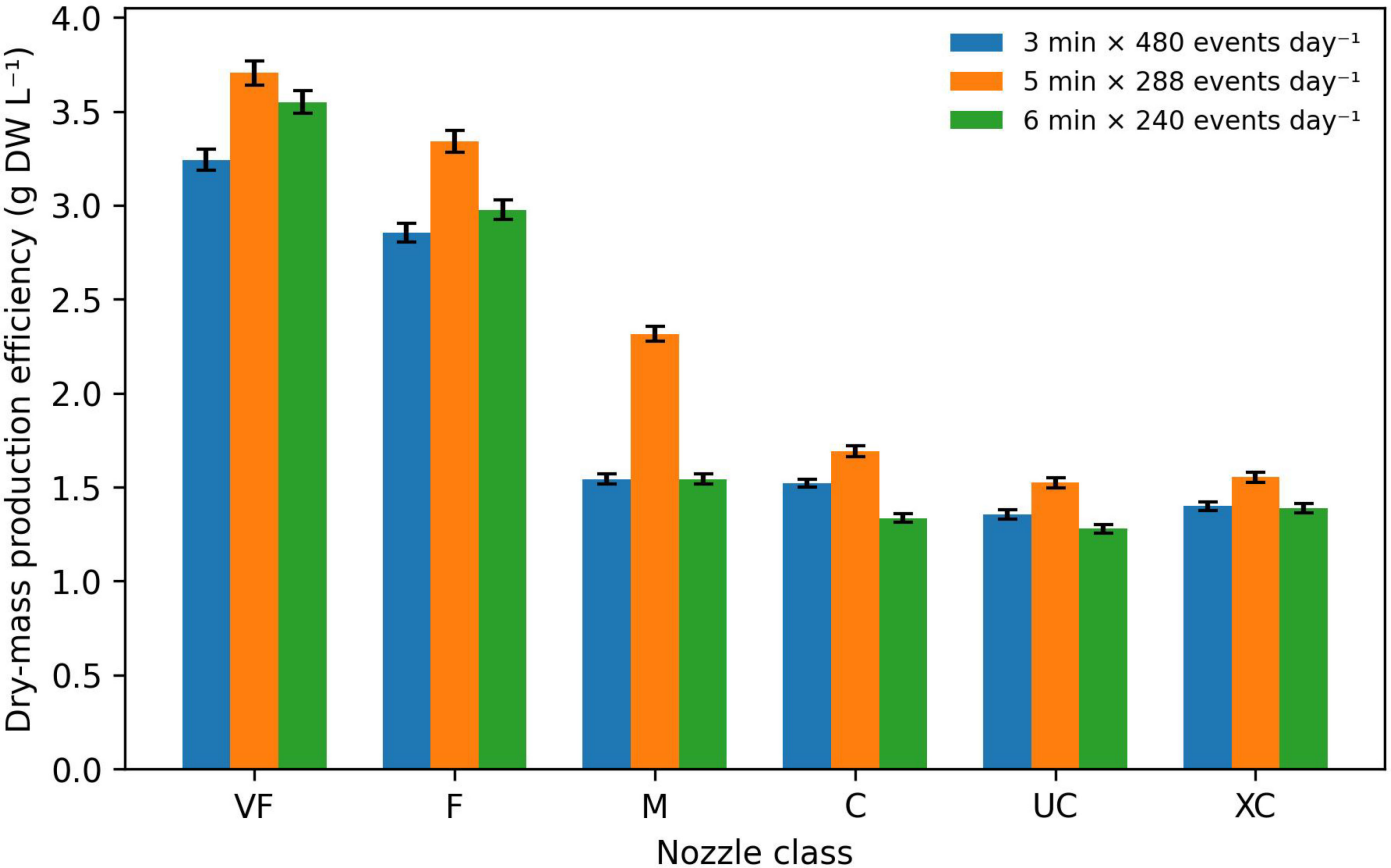
Dry-mass production efficiency of *C. asiatica* across nozzle classes and misting schedules.

**Figure 3:**
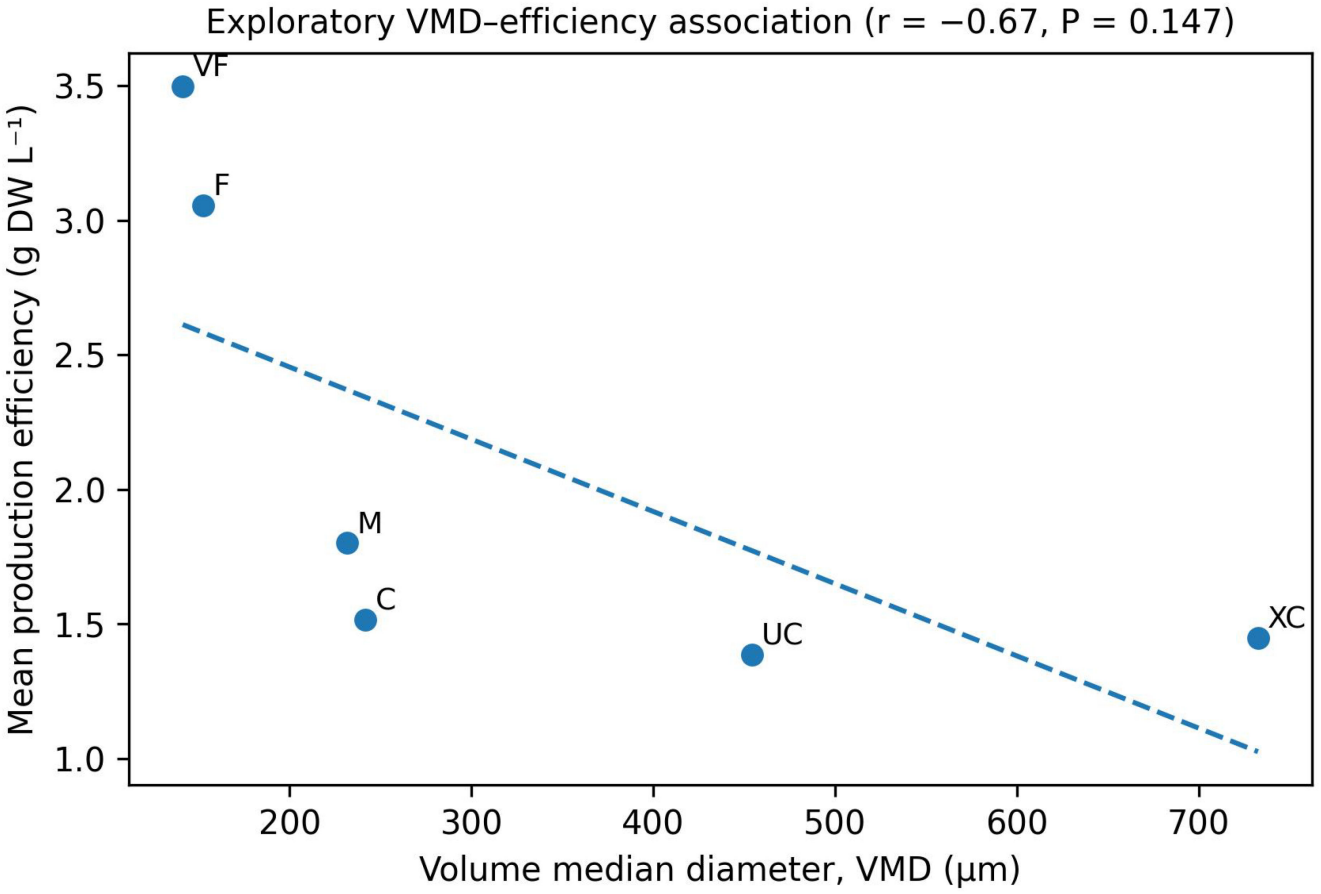
Exploratory association between VMD and mean dry-mass production efficiency across six nozzle classes. Across the six nozzle-level means, VMD was negatively associated with mean production efficiency (r = −0.67, P = 0.147); the same direction was observed within the individual misting schedules. Because the analysis was based on six aggregated nozzle means, it is presented as exploratory rather than confirmatory evidence.

Nozzle class and misting schedule each affected production efficiency (both P < 0.001). The interaction term was retained because interpretation depended on the combined delivery treatment and daily misting volume. In the six-nozzle screen, VF and F had the highest efficiency, M was intermediate, and C, UC, and XC were lower. The subsequent bed-area trial evaluated only the operationally feasible C, M, F, and VF subset (Section 3.2). Across all six nozzle classes, the 5 min × 288 events day^−^¹ schedule produced the highest efficiency. These findings characterize integrated root-zone delivery treatments and do not identify droplet diameter as the sole causal factor. The physical properties of the nozzle treatments and production-efficiency results are summarized in **Table 3** and **Figure 2**. The exploratory association between VMD and mean dry-mass production efficiency is shown in **Figure 3**.

**Table 3:** Physical properties of the nozzle treatments and production efficiency (g DW L^−^¹) under three misting schedules.

Nozzle	SMD (µm)	VMD (µm)	Flow (L min^−^¹)	Angle (°)	3 min × 480 events day^−^¹	5 min × 288 events day^−^¹	6 min × 240 events day^−^¹	Overall mean
VF	115.3	141.2	0.7	108	3.241 ± 0.056	3.704 ± 0.064	3.549 ± 0.061	3.498 ± 0.074 a
F	122.5	152.5	0.6	116	2.853 ± 0.050	3.340 ± 0.058	2.976 ± 0.051	3.056 ± 0.078 b
M	179.5	231.6	0.7	95	1.543 ± 0.027	2.315 ± 0.040	1.542 ± 0.027	1.800 ± 0.130 c
C	184.1	241.6	0.7	125	1.521 ± 0.020	1.692 ± 0.029	1.336 ± 0.023	1.516 ± 0.053 cd
XC	647.9	732.7	0.6	140	1.399 ± 0.024	1.553 ± 0.027	1.389 ± 0.024	1.447 ± 0.029 d
UC	362.2	454.2	0.6	120	1.355 ± 0.024	1.523 ± 0.027	1.279 ± 0.022	1.386 ± 0.038 d

Values are means ± SE (n = 3 per nozzle × schedule combination). Letters in the overall-mean column indicate Tukey HSD groupings across nozzle classes.

### 3.2 Bed-area biomass differed among operational nozzle treatments

**Figure 4:**
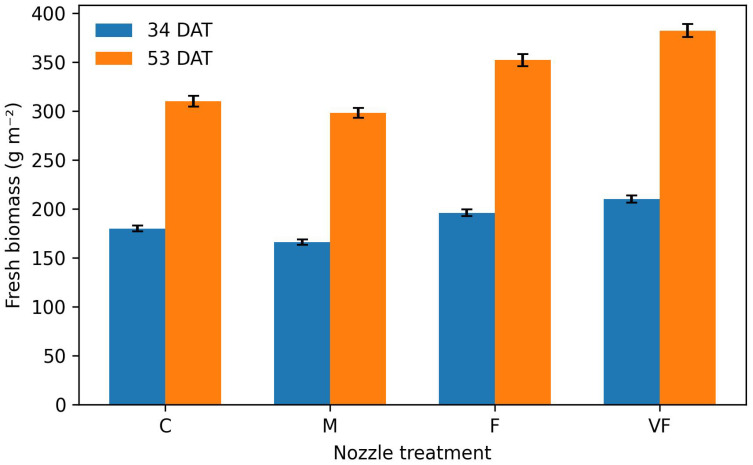
Bed-area fresh biomass at 34 and 53 DAT across nozzle treatments.

**Figure 5:**
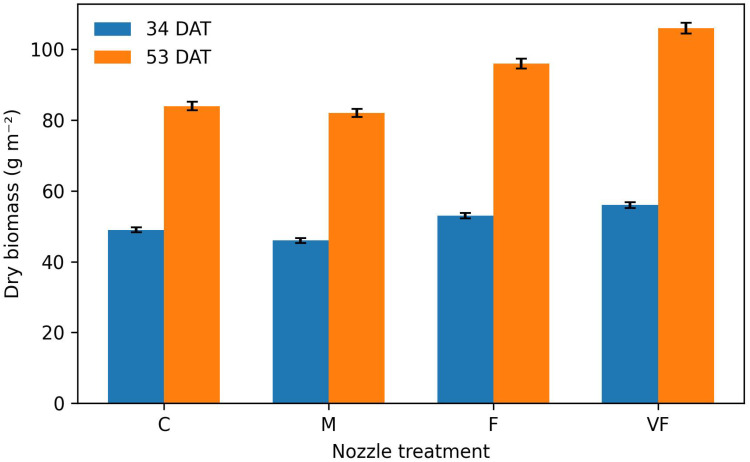
Bed-area dry biomass at 34 and 53 DAT across nozzle treatments. Across the four nozzle-level means, VMD was negatively correlated with 53 DAT fresh biomass (r = −0.95, P = 0.048) and showed a similar association with dry biomass (r = −0.95, P = 0.052). Given the small number of nozzle-level means (n = 4), these correlations are exploratory; the bed-level comparisons provide the primary evidence that F and VF produced more biomass than C and M under the tested conditions.

At both 34 and 53 DAT, fresh and dry biomass differed among nozzle treatments (all P < 0.001). Treatment separation was greater at 53 DAT, when VF produced the highest bed-area fresh and dry biomass, followed by F, whereas C and M were lower. Because plant subsamples were averaged within beds before analysis, these statistics represent bed-level treatment effects rather than individual-plant effects. The bed-area biomass data are summarized in **Table 4** and illustrated in **Figures 4** and **5**.

**Table 4:** Bed-area fresh and dry biomass of *C. asiatica* under four operational nozzle treatments.

DAT	Nozzle	VMD (µm)	Fresh biomass (g m^−^²)	Dry biomass (g m^−^²)	Dry matter (%)	Daily FW gain (g m^−^² d^−^¹)
34	C	241.6	180.0 ± 3.1 c	49.0 ± 0.7 b	27.2 ± 0.1	n.a.
34	M	231.6	166.0 ± 2.9 d	46.0 ± 0.7 b	27.7 ± 0.1	n.a.
34	F	152.5	196.0 ± 3.4 b	53.0 ± 0.7 a	27.0 ± 0.1	n.a.
34	VF	141.2	210.0 ± 3.7 a	56.0 ± 0.8 a	26.7 ± 0.1	n.a.
53	C	241.6	310.0 ± 5.4 c	84.0 ± 1.2 c	27.1 ± 0.1	6.85 ± 0.28
53	M	231.6	298.0 ± 5.2 c	82.0 ± 1.1 c	27.5 ± 0.1	6.95 ± 0.28
53	F	152.5	352.0 ± 6.1 b	96.0 ± 1.4 b	27.3 ± 0.1	8.22 ± 0.33
53	VF	141.2	382.0 ± 6.7 a	106.0 ± 1.5 a	27.8 ± 0.1	9.10 ± 0.35

Values are means ± SE (n = 4 bed-level replicates). Within each DAT and response variable, different letters indicate Tukey HSD differences at P < 0.05; letters are shown for fresh and dry biomass.

### 3.3 Root-zone conditions remained stable under the 5 min × 288 events day^−^¹ schedule

Across nozzle treatments and sampling dates, root-zone temperature ranged from 22.1 to 22.7 °C, root-zone RH from 94.5% to 96.5%, line pressure from 3.98 to 4.03 bar, reservoir EC from 1.27 to 1.83 dS m^−^¹, and pH from 5.92 to 6.05. Mean root-zone RH was 96.3% under C and 94.7% under VF, indicating that VF did not coincide with a more saturated chamber. Across the four nozzle-level means, RH and VMD were positively associated (r = 0.98, P = 0.019); this relationship is descriptive because the sampling dates were not independent environmental replicates. The corresponding root-zone and reservoir conditions are summarized in **Table 5**.

**Table 5:** Root-zone and reservoir conditions during operation under the 5 min × 288 events day^−^¹ schedule.

Nozzle treatment	Root-zone temperature (°C)	Root-zone RH (%)	Line pressure (bar)	Reservoir EC (dS m^−^¹)	Reservoir pH
C	22.30 ± 0.09	96.30 ± 0.09	4.01 ± 0.00	1.58 ± 0.11	6.01 ± 0.02
M	22.35 ± 0.12	95.80 ± 0.09	4.00 ± 0.00	1.60 ± 0.11	5.99 ± 0.02
F	22.40 ± 0.09	94.90 ± 0.09	3.98 ± 0.00	1.63 ± 0.11	5.97 ± 0.02
VF	22.45 ± 0.12	94.70 ± 0.09	4.03 ± 0.00	1.64 ± 0.11	5.96 ± 0.02

Values are descriptive means ± SE across four stage-level monitoring dates (7, 21, 42, and 53 DAT); dates were not independent treatment replicates.

### 3.4 Stage-specific ion management improved whole-plant growth and solution-use efficiency

**Figure 6:**
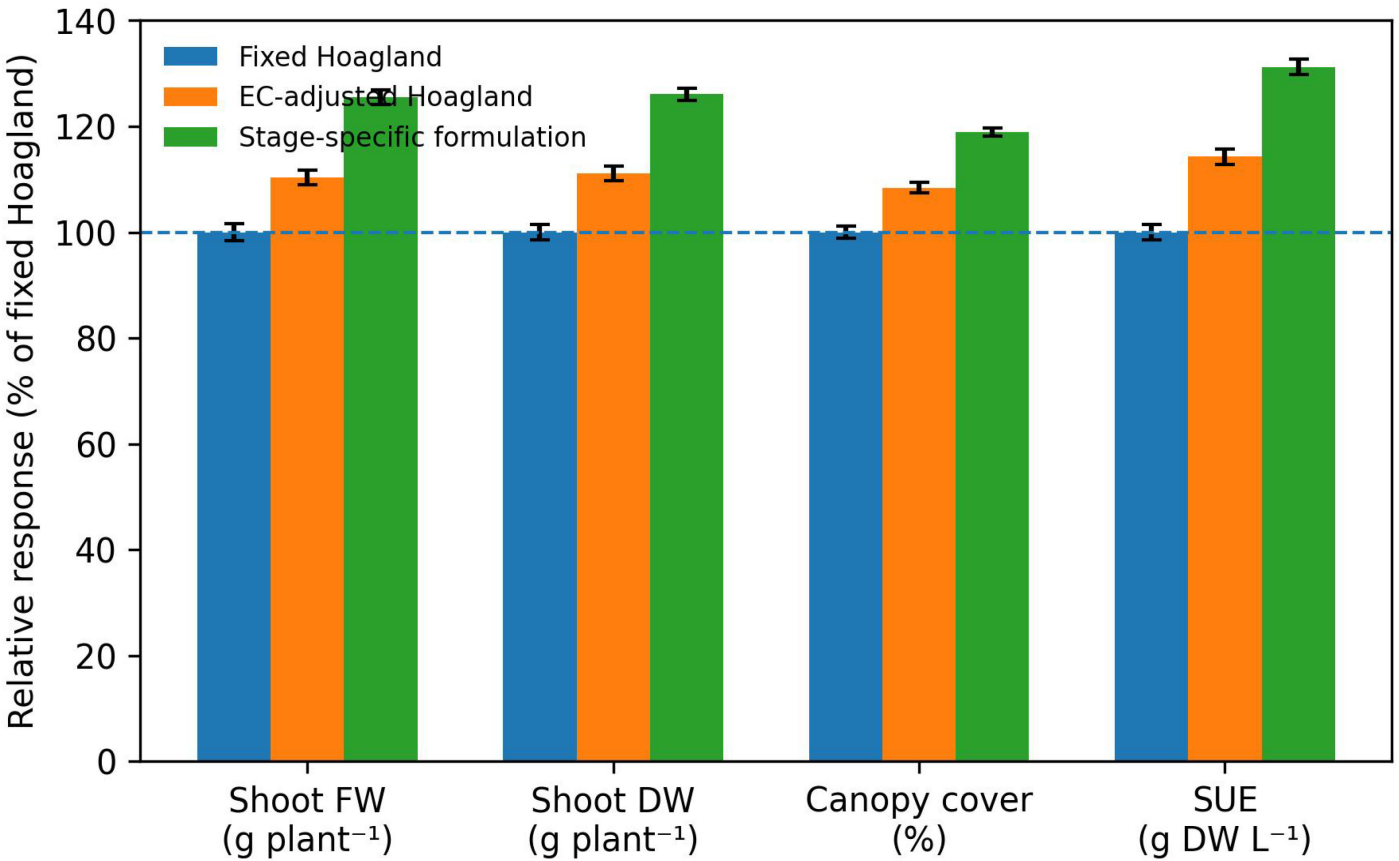
Relative plant-growth and solution-use responses to nutrient-management treatments.

The stage-specific formulation produced higher values for all measured traits than either fixed or EC-adjusted Hoagland. Relative to fixed Hoagland, the stage-specific formulation increased shoot fresh weight by 25.6%, shoot dry weight by 26.1%, leaf number by 26.9%, runner number by 36.4%, root dry weight by 24.2%, SPAD by 8.7%, canopy cover by 18.9%, and solution-use efficiency by 31.3%. All variables showed significant treatment effects by one-way ANOVA followed by Tukey HSD. The plant-growth and resource-use responses are summarized in **Table 6** and illustrated in **Figure 6**.

**Table 6:** Plant-growth and resource-use responses to nutrient-management treatment.

Variable	Fixed Hoagland	EC-adjusted Hoagland	Stage-specific formulation	Change vs fixed	ANOVA
Shoot FW (g plant^−^¹)	81.30 ± 1.34 c	89.70 ± 1.13 b	102.10 ± 1.13 a	+25.6%	P < 0.001
Shoot DW (g plant^−^¹)	9.00 ± 0.13 c	10.00 ± 0.13 b	11.35 ± 0.10 a	+26.1%	P < 0.001
Leaf number	33.5 ± 0.6 c	37.0 ± 0.4 b	42.5 ± 0.6 a	+26.9%	P < 0.001
Runner number	5.90 ± 0.13 c	6.80 ± 0.13 b	8.05 ± 0.10 a	+36.4%	P < 0.001
Root DW (g plant^−^¹)	1.40 ± 0.02 c	1.53 ± 0.02 b	1.73 ± 0.02 a	+24.2%	P < 0.001
SPAD	39.45 ± 0.31 c	41.15 ± 0.27 b	42.90 ± 0.26 a	+8.7%	P < 0.001
Canopy cover (%)	71.25 ± 0.78 c	77.25 ± 0.69 b	84.75 ± 0.57 a	+18.9%	P < 0.001
SUE (g DW L^−^¹)	1.12 ± 0.02 c	1.28 ± 0.02 b	1.47 ± 0.02 a	+31.3%	P < 0.001

Values are means ± SE (n = 4 bed-level replicates). Different letters within a row indicate Tukey HSD differences at P < 0.05.

### 3.5 Stage-specific reservoir correction requirements differed among crop stages

**Figure 7:**
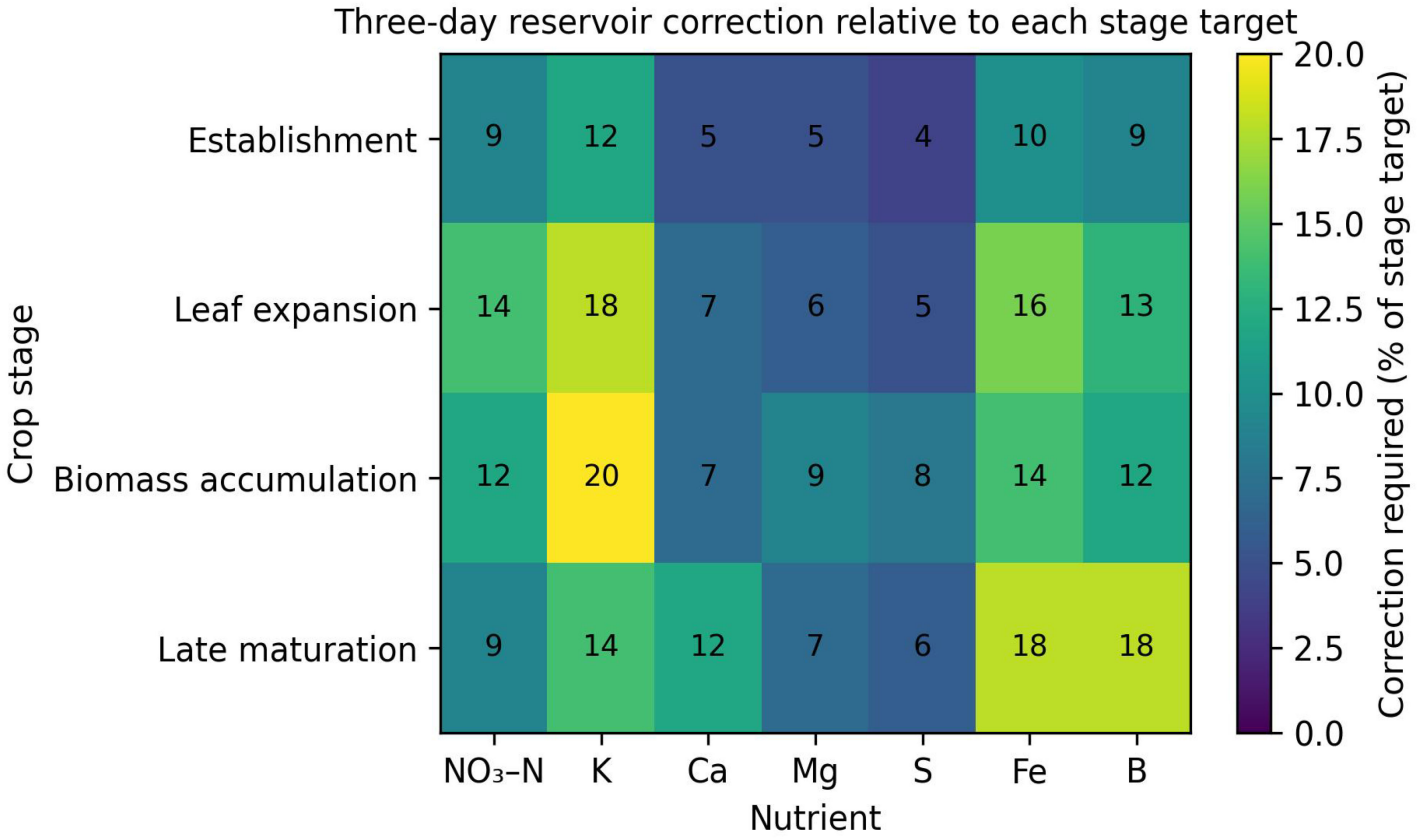
Three-day reservoir correction requirement as a percentage of each stage-specific nutrient target. Each cell equals 100 × [(target after replenishment − concentration before replenishment)/target after replenishment]. Rows indicate crop stages, columns indicate nutrients, and darker shading indicates a larger percentage correction. Absolute targets, pre-replenishment concentrations, and correction values, including NH_4_–N and P, are provided in **Supplementary Table 4**. For example, the 20% K correction during biomass accumulation corresponds to 61.0 mg L^−^¹ relative to a 305 mg L^−^¹ target. Percentages reflect reservoir replenishment, not tissue accumulation.

Normalized 3-day reservoir corrections differed among crop stages (**Figure 7**). The K correction was greatest during biomass accumulation (61.0 mg L^−^¹; 20% of the stage target), whereas the nitrate correction was greatest during leaf expansion (25.62 mg N L^−^¹; 14%). During late maturation, relative corrections were higher for Ca (12%), Fe (18%), and B (18%) than during earlier stages. These values represent reservoir replenishment requirements rather than direct plant uptake.

### 3.6 Nozzle maintenance observations

Field maintenance records indicated a trade-off between fine-droplet operation and clogging risk. The smallest nozzle class clogged approximately once per crop cycle under the tested filtration and operating conditions, whereas larger nozzle classes showed little or no clogging. Because these observations were not obtained in a replicated maintenance experiment, they are reported descriptively and used only to inform the operating protocol. The descriptive clogging observations and recommended operating responses are summarized in **Table 7**.

**Table 7:** Descriptive field observations of nozzle clogging and recommended maintenance.

Nozzle class	Clogging observation	Recommended operating response
Very fine/fine (VF, F)	Highest observed risk; the smallest nozzle clogged approximately once per crop cycle	Use fine filtration, inspect spray pattern at least once per crop cycle, and clean immediately when flow or pattern changes
Medium/coarse (M, C)	Rare under the tested conditions	Maintain routine filter checks and periodic pressure monitoring
Very coarse/extremely coarse (UC, XC)	No or minimal clogging observed	Lower clogging risk, but production efficiency and operational suitability were lower in this study

## 4 Discussion

### 4.1 Root-zone delivery as a plant water–nutrient treatment

By treating nozzle properties as descriptors of the root-zone environment, this study connects an operational variable to plant responses. Aeroponic roots must receive water and dissolved ions while retaining sufficient gas exchange; delivery uniformity and timing can therefore affect growth even when bulk-solution chemistry is adequate (Blok et al., 2017; Eldridge et al., 2020). The observed responses in dry-mass production, shoot and root biomass, canopy development, SPAD, and solution-use efficiency are accordingly interpreted as integrated water–nutrient–oxygen responses rather than as engineering performance alone. Comparable effects of cultivation system, atomizer, and spray interval on root morphology, nutrient uptake, photosynthetic performance, and phytochemical traits have been reported in lettuce (Li et al., 2018; Lakhiar et al., 2019; Tunio et al., 2021, 2022).

### 4.2 Integrated responses to fine-droplet delivery

VF and F combined the smallest SMD and VMD values with the highest shoot dry-mass production efficiency. However, nozzle classes also differed in flow rate, spray angle, spatial distribution, and clogging risk. The data therefore support an integrated fine-droplet delivery treatment effect under the tested conditions, not a universal rule that decreasing droplet diameter alone increases biomass. Greater root-surface coverage and contact frequency are plausible explanations, but neither was measured directly (Blok et al., 2017; Eldridge et al., 2020).

The 5 min × 288 events day^−^¹ schedule produced the highest efficiency within every nozzle class, indicating that delivery timing contributed to the plant response. An intermediate schedule may reduce root-surface drying without maintaining a persistent liquid film that restricts gas transfer. Mean chamber RH did not increase under VF, but RH cannot resolve root-surface wetness, root respiration, or oxygen availability. Direct measurements of root-zone oxygen, root-surface water status, root respiration, and tissue ion accumulation are therefore needed to test the proposed mechanism.

### 4.3 Bed-area biomass corroborated the screening response

The bed-area experiment showed that greater solution-use efficiency under F and VF coincided with greater canopy biomass accumulation. Dry mass per liter and biomass per bed area address different questions: the first describes dry-mass output per unit spray-volume index, whereas the second describes production per cultivation area. Previous aeroponic studies likewise found that cultivation system, atomizer, and delivery interval can modify root and shoot development (Li et al., 2018; Tunio et al., 2021, 2022). The agreement between these two response metrics strengthens the interpretation of a coordinated plant-growth response within the tested nozzle range.

Because UC and XC were not included in the bed-area experiment, inference is limited to the operational C–M–F–VF range. Their exclusion was based on the screening results and operational constraints; consequently, the bed-area data cannot establish a continuous response across the full droplet-size spectrum.

### 4.4 Stage-specific ion management linked reservoir correction with plant development

The stage-specific formulation produced coordinated improvements in shoot and root biomass, leaf and runner number, canopy cover, SPAD, and solution-use efficiency relative to fixed and EC-adjusted Hoagland. These responses support the principle that maintaining bulk EC does not ensure that ionic composition matches developmental demand. Plant-demand-based nutrient management has improved water and nutrient use in other closed soilless crops (Signore et al., 2016), and ion-ratio monitoring can reveal composition drift that EC alone cannot resolve (Gang et al., 2025).

**Figure 7** indicates distinct stage-specific reservoir corrections: K was most prominent during biomass accumulation, nitrate during leaf expansion, and Ca, Fe, and B during late maturation. This sequence is physiologically plausible given the roles of K in osmotic regulation and assimilate transport, nitrate in leaf expansion and protein synthesis, Ca and B in cell-wall and meristem stability, and Fe in photosynthetic electron transport and chlorophyll metabolism (Brown et al., 2002; White and Broadley, 2003; Marschner, 2012; Wang et al., 2013; Kobayashi et al., 2019). Nevertheless, reservoir correction is a net system-level quantity rather than a direct measure of uptake; tissue mineral analysis and reservoir mass balance are needed to confirm element-specific uptake kinetics.

### 4.5 Commercial implications and area basis

Bed-area and whole-greenhouse productivity should be distinguished. The crop-supporting surface occupied 63.9 m² of the 165 m² greenhouse floor area (38.7%). Bed-area values are therefore appropriate for treatment comparisons, whereas commercial productivity estimates must also account for aisles, equipment, service space, and bed layout.

For commercial deployment, the productivity advantage of fine nozzles must be balanced against their higher maintenance burden. Fine filtration, scheduled cleaning, spray-pattern verification, and pressure monitoring should therefore accompany VF or F operation (Min et al., 2023).

Because the experiments used natural sunlight transmitted through a fluorine-film greenhouse, seasonal radiation may confound comparisons among crop cycles. Internal DLI ranged from 2–7 mol m^−^² day^−^¹ in winter to 14–22 mol m^−^² day^−^¹ during the June high-light period. Future trials should record canopy-level light continuously and include cumulative DLI or PAR as a covariate or blocking factor (Both et al., 2015; Marcelis et al., 2006; Faust and Logan, 2018).

### 4.6 Limitations and priorities for physiological validation

Nozzle class, misting schedule, and nutrient formulation were evaluated in complementary experiments rather than in one complete factorial design. Their interactions therefore cannot be estimated from the present dataset. A confirmatory design should combine these factors, randomize bed allocation, repeat crop cycles, and account for DLI or season through blocking or covariate analysis.

Nozzle class also bundled several physical variables, including droplet spectrum, flow distribution, local wetting pattern, spray angle, and maintenance risk. Moreover, the study did not directly measure root-surface wetness, gas-phase or dissolved oxygen, root respiration, plant water status, root-system architecture, photosynthetic gas exchange, or tissue ion concentrations. These measurements are needed to connect the observed biomass and SPAD responses to specific root physiological processes. Filtration performance and clogging should also be incorporated into future treatment evaluation.

A further limitation concerns medicinal-crop quality. The present study prioritized whole-plant growth and resource-use traits, whereas *C. asiatica* is valued for madecassoside and asiaticoside. Future experiments should retain these compounds as secondary quality endpoints even when biomass is the primary response, thereby avoiding optimization of growth at the expense of product quality.

## 5 Conclusion

This study associates integrated root-zone droplet-delivery treatments and stage-specific ion management with growth and nutrient-solution-use efficiency in aeroponic *C. asiatica*. VF and F were the most efficient nozzle classes, the 5 min × 288 events day^−^¹ schedule was the most efficient schedule, and VF produced the greatest 53 DAT bed-area biomass among four operational nozzle treatments. A stage-specific nutrient formulation improved shoot and root growth and solution-use efficiency relative to fixed or EC-adjusted Hoagland. These findings support coordinated management of physical root-zone delivery and ionic composition, while mechanistic attribution to droplet size, root oxygen status, or element uptake requires multi-season factorial experiments with direct physiological measurements.

## Data Availability

The raw data supporting the conclusions of this article will be made available by the authors, without undue reservation.
