# Supplementary material for: Fine droplet misting and stage-specific nutrient formulation enhance bed-area biomass productivity and nutrient solution use efficiency in closed-loop aeroponic Centella asiatica
Source: Front Plant Sci. 2026 Jul 23;17:1892738. doi: 10.3389/fpls.2026.1892738 (PMC13442833; doi:10.3389/fpls.2026.1892738)
Supplement: Supplementary file 1 [file Table1.docx]

# Supplementary material included in this submission

Supplementary Tables S1–S4 provide the core datasets used in the analyses, with each title placed immediately before its corresponding table. Machine readable versions accompany the submission.

**Supplementary Table S1. Nutrient-management validation raw dataset.**

| **Treatment** | **Rep** | **Shoot FW** | **Shoot DW** | **Leaves** | **Runners** | **Root DW** | **SPAD** | **Canopy %** | **SUE** |
| --- | --- | --- | --- | --- | --- | --- | --- | --- | --- |
| Fixed Hoagland | 1 | 78.1 | 8.7 | 32 | 5.6 | 1.35 | 38.7 | 69.4 | 1.08 |
| Fixed Hoagland | 2 | 84.5 | 9.3 | 35 | 6.2 | 1.44 | 40.2 | 73.1 | 1.16 |
| Fixed Hoagland | 3 | 80.6 | 8.9 | 33 | 5.8 | 1.39 | 39.3 | 70.8 | 1.11 |
| Fixed Hoagland | 4 | 82.0 | 9.1 | 34 | 6.0 | 1.41 | 39.6 | 71.7 | 1.13 |
| EC-adjusted | 1 | 87.0 | 9.7 | 36 | 6.5 | 1.48 | 40.5 | 75.6 | 1.24 |
| EC-adjusted | 2 | 92.4 | 10.3 | 38 | 7.1 | 1.58 | 41.8 | 78.9 | 1.32 |
| EC-adjusted | 3 | 89.1 | 9.9 | 37 | 6.7 | 1.52 | 41.0 | 76.9 | 1.27 |
| EC-adjusted | 4 | 90.3 | 10.1 | 37 | 6.9 | 1.54 | 41.3 | 77.6 | 1.29 |
| Dynamic formulation | 1 | 99.4 | 11.1 | 41 | 7.8 | 1.68 | 42.3 | 83.4 | 1.43 |
| Dynamic formulation | 2 | 104.8 | 11.6 | 44 | 8.3 | 1.79 | 43.5 | 86.1 | 1.51 |
| Dynamic formulation | 3 | 101.5 | 11.3 | 42 | 8.0 | 1.72 | 42.7 | 84.4 | 1.46 |
| Dynamic formulation | 4 | 102.7 | 11.4 | 43 | 8.1 | 1.75 | 43.1 | 85.1 | 1.48 |

**Supplementary Table S2. Bed area biomass raw dataset.**

| **DAT** | **Nozzle** | **Rep** | **Fresh biomass (g m⁻²)** | **Dry biomass (g m⁻²)** | **Dry matter (%)** | **Daily FW gain (g m⁻² d⁻¹)** |
| --- | --- | --- | --- | --- | --- | --- |
| 34 | C | 1 | 172.8 | 47.4 | 27.4 |  |
| 34 | C | 2 | 177.3 | 48.4 | 27.3 |  |
| 34 | C | 3 | 182.7 | 49.6 | 27.1 |  |
| 34 | C | 4 | 187.2 | 50.6 | 27.0 |  |
| 53 | C | 1 | 322.4 | 86.7 | 26.9 | 7.5 |
| 53 | C | 2 | 314.6 | 85.0 | 27.0 | 7.1 |
| 53 | C | 3 | 305.4 | 83.0 | 27.2 | 6.6 |
| 53 | C | 4 | 297.6 | 81.3 | 27.3 | 6.2 |
| 34 | M | 1 | 159.4 | 44.5 | 27.9 |  |
| 34 | M | 2 | 163.5 | 45.4 | 27.8 |  |
| 34 | M | 3 | 168.5 | 46.6 | 27.7 |  |
| 34 | M | 4 | 172.6 | 47.5 | 27.5 |  |
| 53 | M | 1 | 309.9 | 84.6 | 27.3 | 7.6 |
| 53 | M | 2 | 302.5 | 83.0 | 27.4 | 7.2 |
| 53 | M | 3 | 293.5 | 81.0 | 27.6 | 6.7 |
| 53 | M | 4 | 286.1 | 79.4 | 27.8 | 6.3 |
| 34 | F | 1 | 188.2 | 51.3 | 27.3 |  |
| 34 | F | 2 | 193.1 | 52.4 | 27.1 |  |
| 34 | F | 3 | 198.9 | 53.6 | 26.9 |  |
| 34 | F | 4 | 203.8 | 54.7 | 26.8 |  |
| 53 | F | 1 | 366.1 | 99.1 | 27.1 | 9.0 |
| 53 | F | 2 | 357.3 | 97.2 | 27.2 | 8.5 |
| 53 | F | 3 | 346.7 | 94.8 | 27.3 | 7.9 |
| 53 | F | 4 | 337.9 | 92.9 | 27.5 | 7.5 |
| 34 | VF | 1 | 201.6 | 54.2 | 26.9 |  |
| 34 | VF | 2 | 206.8 | 55.3 | 26.7 |  |
| 34 | VF | 3 | 213.1 | 56.7 | 26.6 |  |
| 34 | VF | 4 | 218.4 | 57.8 | 26.5 |  |
| 53 | VF | 1 | 397.3 | 109.4 | 27.5 | 9.9 |
| 53 | VF | 2 | 387.7 | 107.3 | 27.7 | 9.4 |
| 53 | VF | 3 | 376.3 | 104.7 | 27.8 | 8.8 |
| 53 | VF | 4 | 366.7 | 102.6 | 28.0 | 8.3 |

**Supplementary Table S3. Root-zone environment monitoring dataset.**

| **DAT** | **Stage** | **Nozzle** | **Root-zone temperature (°C)** | **Root-zone RH (%)** | **Line pressure (bar)** | **Reservoir EC (dS m⁻¹)** | **Reservoir pH** |
| --- | --- | --- | --- | --- | --- | --- | --- |
| 7 | Establishment | C | 22.10 | 96.10 | 4.01 | 1.27 | 6.05 |
| 7 | Establishment | M | 22.10 | 95.60 | 4.00 | 1.29 | 6.03 |
| 7 | Establishment | F | 22.20 | 94.70 | 3.98 | 1.32 | 6.01 |
| 7 | Establishment | VF | 22.20 | 94.50 | 4.03 | 1.33 | 6.00 |
| 21 | Leaf expansion | C | 22.20 | 96.20 | 4.01 | 1.67 | 6.05 |
| 21 | Leaf expansion | M | 22.20 | 95.70 | 4.00 | 1.69 | 6.03 |
| 21 | Leaf expansion | F | 22.30 | 94.80 | 3.98 | 1.72 | 6.01 |
| 21 | Leaf expansion | VF | 22.30 | 94.60 | 4.03 | 1.73 | 6.00 |
| 42 | Biomass accumulation | C | 22.40 | 96.40 | 4.01 | 1.77 | 5.97 |
| 42 | Biomass accumulation | M | 22.50 | 95.90 | 4.00 | 1.79 | 5.95 |
| 42 | Biomass accumulation | F | 22.50 | 95.00 | 3.98 | 1.82 | 5.93 |
| 42 | Biomass accumulation | VF | 22.60 | 94.80 | 4.03 | 1.83 | 5.92 |
| 53 | Production endpoint | C | 22.50 | 96.50 | 4.01 | 1.62 | 5.97 |
| 53 | Production endpoint | M | 22.60 | 96.00 | 4.00 | 1.64 | 5.95 |
| 53 | Production endpoint | F | 22.60 | 95.10 | 3.98 | 1.67 | 5.93 |
| 53 | Production endpoint | VF | 22.70 | 94.90 | 4.03 | 1.68 | 5.92 |

**Supplementary Table S4. Full stage-specific nutrient target, before replenishment, and calculated correction dataset.**

| **Stage** | **Sampling point** | **Day** | **NO₃–N** | **NH₄–N** | **P** | **K** | **Ca** | **Mg** | **S** | **Fe** | **B** |
| --- | --- | --- | --- | --- | --- | --- | --- | --- | --- | --- | --- |
| Establishment | Target after replenishment | 0 | 145.00 | 3.00 | 28.00 | 190.00 | 160.00 | 38.00 | 62.00 | 2.00 | 0.33 |
| Establishment | Before replenishment | 3 | 131.95 | 2.76 | 26.32 | 167.20 | 152.00 | 36.10 | 59.52 | 1.80 | 0.30 |
| Establishment | Calculated correction | 3 | 13.05 | 0.24 | 1.68 | 22.80 | 8.00 | 1.90 | 2.48 | 0.20 | 0.03 |
| Leaf expansion | Target after replenishment | 0 | 183.00 | 3.00 | 33.00 | 265.00 | 180.00 | 45.00 | 72.00 | 2.50 | 0.45 |
| Leaf expansion | Before replenishment | 3 | 157.38 | 2.76 | 30.69 | 217.30 | 167.40 | 42.30 | 68.40 | 2.10 | 0.39 |
| Leaf expansion | Calculated correction | 3 | 25.62 | 0.24 | 2.31 | 47.70 | 12.60 | 2.70 | 3.60 | 0.40 | 0.06 |
| Biomass accumulation | Target after replenishment | 0 | 168.00 | 3.00 | 30.00 | 305.00 | 190.00 | 52.00 | 78.00 | 2.50 | 0.50 |
| Biomass accumulation | Before replenishment | 3 | 147.84 | 2.76 | 28.20 | 244.00 | 176.70 | 47.32 | 71.76 | 2.15 | 0.44 |
| Biomass accumulation | Calculated correction | 3 | 20.16 | 0.24 | 1.80 | 61.00 | 13.30 | 4.68 | 6.24 | 0.35 | 0.06 |
| Late maturation | Target after replenishment | 0 | 148.00 | 2.00 | 28.00 | 255.00 | 210.00 | 48.00 | 72.00 | 3.00 | 0.60 |
| Late maturation | Before replenishment | 3 | 134.68 | 1.90 | 26.32 | 219.30 | 184.80 | 44.64 | 67.68 | 2.46 | 0.49 |
| Late maturation | Calculated correction | 3 | 13.32 | 0.10 | 1.68 | 35.70 | 25.20 | 3.36 | 4.32 | 0.54 | 0.11 |

Mn, Zn, Cu, and Mo were also recorded but are omitted from this compact table to preserve readability.
